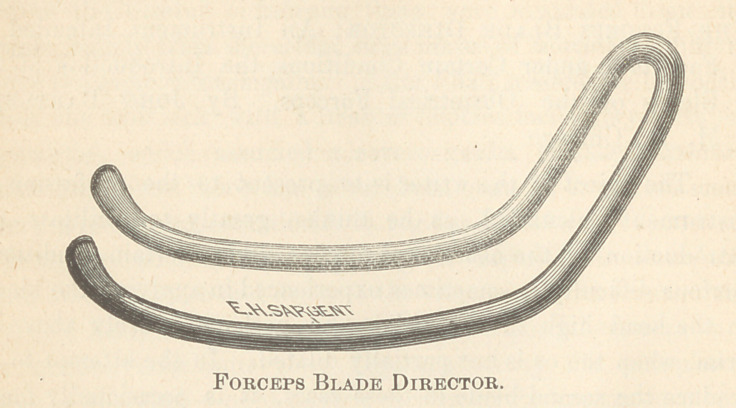# The Forceps Blade Director; an Instrument Intended to Facilitate the Introduction of the Blades of the Obstetrical Forceps

**Published:** 1882-04

**Authors:** John Bartlett

**Affiliations:** Chicago


ARTICLE III.

THE FORCEPS BLADE DIRECTOR; An Instrument Intended to Facilitate, under Certain Conditions, the Introduction of the Blades of the Obstetrical Forceps. By JOHN BARTLETT, M. D., Chicago.

The object of the writer is to present to the profession an instrument calculated, as he thinks, greatly to facilitate the introduction of the obstetrical forceps under certain conditions. Serious difficulty is sometimes experienced in applying the blades to the head high in the pelvis, or perhaps entirely above its brim, when the os is but partially dilated. In the attempt to introduce the second blade in these cases, it is occasionally found that the right margin of the os, because of the tension made on the opposite circumference of the opening by the shank of the blade already in position, has been drawn to the left quite beyond the median line. So that a blade, introduced into the os with difficulty, is estopped from gliding into its true position by impinging against the *left* plane of the presenting globe of the head, and is invited by this plane to pass on to the left side of the pelvis in company with the blade already introduced. To overcome this difficulty it is necessary in some way, by the fingers or the point of the blade, to draw or push the displaced circumference of the os so far toward its proper position on the right as to permit of the point of the blade's reaching and gliding upon the right plane of the globe of the head. The maneuvers necessary to accomplish this end are especially difficult when the vagina is narrow, the head high, and the os uteri small. For under such circumstances, the fingers serving as directors of the blade and retractors of the os fall short, and are frequently unable to retain their position at the mouth of the womb, at the same instant that the blade is pushed upward to enter it. As a result of these difficulties the practitioner, vainly resorting to the use of the fingers of one, and the other hand, presented now with the dorsal and now with the palmar surface toward the point desired to be reached, and inserting, perhaps hurriedly, now the half hand and now the fingers only, too often harasses the patient, alarms the friends, and embarrasses himself with a series of failures before attaining success.

FORCEPS BLADE DIRECTOR.

To obviate these difficulties, an instrument which may be designated the forceps blade director, is here presented. It is made of iron wire, less than a quarter of an inch in diameter, formed at first into the shape of a hair-pin with the rods parallel. These are cut off at suitable length and are given, edgewise, as regards their plane, a vaginal curve. The ends are then flattened out and gently curved away from the plane of the rods, as retractors. The opposite extremity of the instrument, at a suitable point, is bent away from the plane of the rods, in the same direction as the retracting extremities, to serve as a handle.

The accompanying wood cut will give a good idea of the instrument, and will at once suggest the mode of using it. The first blade resting in place, the retractor, held gently by the handle in the left hand, its rods resting, the one above, the other below the middle finger of the right hand, is inserted into the vagina, and guided so that the points enter the os uteri and are brought to bear upon its right margin. The fingers are then withdrawn, while the instrument is steadied in place by the left hand still grasping the handle (purposely bent away from the median line) in such manner as to retract the os uteri toward the right side and to draw it slightly away from the head. The instrument now serves as a guide to the forceps blade, the rounded point of which is glided along the "ways" formed by the rods directly into the os, and between it and the right plane of the head. One or the other retracting point of the director now falls within the fenestra of the blade, leaving its rims and the rods of the retractor in different planes, so that the latter are free to be withdrawn without disturbing the position of the former.

The contrivance, it will be seen, is, at once, an os uteri retractor and a forceps blade director. The instrument to which reference has been made, so far, is adapted for use in introducing the second blade only; there is a companion to it, similar, *mutatis mutandis*, for the left side, though occasions for the use of this director will be rare.

---

HOW TO CONTRIBUTE TO MEDICAL JOURNALS.—If you desire to prepare valuable communications for medical journals, you must observe the following rules:

1. Be positively sure you have something *new* to contribute.
2. Tell it in as few words as possible.
3. Do not plagiarize from text-books.
4. Never report an uncertain case without a verification or falsification of the diagnosis by a post-mortem examination.
5. Do not send the same communication to half a dozen journals. Select one, and send it to no others. If you neglect this rule, your habit will ultimately be found out, and *all* journals will reject your communications.—*Med. and Surg. Reporter*;

DR. WM. S. JANNEY, Coroner of Philadelphia, reports the following death-roll from his department for 1881: A list of 1,915 cases shows that 108 persons died during the year without leaving a single clue to establish their identity. Of this number seven were infants abandoned at birth; 38 of the unknown were men and three were women.—*Med. Bulletin.*

THE *Glasgow Medical Journal* contributes two cases of poisoning following the use of iodoform dressing in carious diseases. One of these had parenchymatous disease of the liver and kidneys.

## Figures and Tables

**Figure f1:**